# Issues in Continuous 24-h Core Body Temperature Monitoring in Humans Using an Ingestible Capsule Telemetric Sensor

**DOI:** 10.3389/fendo.2017.00130

**Published:** 2017-06-13

**Authors:** Cathriona R. Monnard, Elie-Jacques Fares, Julie Calonne, Jennifer L. Miles-Chan, Jean-Pierre Montani, Dominique Durrer, Yves Schutz, Abdul G. Dulloo

**Affiliations:** ^1^Division of Physiology, Department of Medicine, University of Fribourg, Fribourg, Switzerland; ^2^Cabinet Médical COM’s, EUROBESITAS, Vevey, Switzerland

**Keywords:** core body temperature, telemetry, thermogenesis, obesity, thrifty metabolism, energy expenditure

## Abstract

**Background:**

There is increasing interest in the use of pill-sized ingestible capsule telemetric sensors for assessing core body temperature (Tc) as a potential indicator of variability in metabolic efficiency and thrifty metabolic traits. The aim of this study was to investigate the feasibility and accuracy of measuring Tc using the CorTemp^®^ system.

**Methods:**

Tc was measured over an average of 20 h in 27 human subjects, with measurements of energy expenditure made in the overnight fasted state at rest, during standardized low-intensity physical activity and after a 600 kcal mixed meal. Validation of accuracy of the capsule sensors was made *ex vivo* against mercury and electronic thermometers across the physiological range (35–40°C) in morning and afternoon of 2 or 3 consecutive days. Comparisons between capsule sensors and thermometers were made using Bland–Altman analysis. Systematic bias, error, and temperature drift over time were assessed.

**Results:**

The circadian Tc profile classically reported in free-living humans was confirmed. Significant increases in Tc (+0.2°C) were found in response to low-power cycling at 40–50 W (~3–4 METs), but no changes in Tc were detectable during low-level isometric leg press exercise (<2 METs) or during the peak postprandial thermogenesis induced by the 600 kcal meal. Issues of particular interest include fast “turbo” gut transit with expulsion time of <15 h after capsule ingestion in one out of every five subjects and sudden erratic readings in teletransmission of Tc. Furthermore, *ex vivo* validation revealed a substantial mean bias (exceeding ±0.5°C) between the Tc capsule readings and mercury or electronic thermometers in half of the capsules. When examined over 2 or 3 days, the initial bias (small or large) drifted in excess of ±0.5°C in one out of every four capsules.

**Conclusion:**

Since Tc is regulated within a very narrow range in the healthy homeotherm’s body (within 1°C), physiological investigations of Tc require great accuracy and precision (better than 0.1°C). Although ingestible capsule methodology appears of great interest for non-invasively monitoring the transit gut temperature, new technology requires a reduction in the inherent error of measurement and elimination of temperature drift and warrants more interlaboratory investigation on the above factors.

## Introduction

During the past decades, there has been considerable debate about the potential role of a low resting energy expenditure (EE) as a “thrifty” metabolic trait in the development of obesity (1, 2) and in the ease with which weight is regained after therapeutic slimming (3). In more recent years, there has been renewed interest in examining a low core (or central) body temperature (Tc) as another potential thrifty metabolic trait (4). As approximately 40% of total EE is used to maintain Tc (referred to as the thermoregulatory component of EE) in human adults (4, 5), it follows that differences or changes in Tc, even of small magnitude, are likely to exert a significant effect on EE. The reported difference in Tc between non-obese and obese, which has been suggested to reflect “thrifty” metabolism, is in the range of 0.1–0.3°C (6, 7). Given the small magnitude of this difference, capsules used to measure Tc should be accurate enough to detect changes within ±0.1°C. Originally designed to measure exercise and occupational heat stress, telemetric Tc ingestible capsules have now been extended for use in the measurement of intestinal Tc in the research setting. Tc capsules offer significant advantages over the traditional measurements of rectal and esophageal temperature, particularly in free-living research participants, and good agreements between telemetric capsules and esophageal and rectal temperature have previously been reported during exercise heat stress (8, 9).

To date, however, Tc comparisons between lean and obese subjects have yielded conflicting results (6, 10–15), including among studies that have measured Tc using ingestible capsule telemetry sensors. Indeed, both a lower (7, 15) and a higher Tc (6) have been reported in overweight/obese subjects compared with lean, while others found no association between Tc and weight or body mass index (BMI) (6, 16). The controversy surrounding the association between Tc and body weight may be due in part to the fact that the reliability and reproducibility of devices for measuring Tc are not well defined.

The aim of this study, therefore, was twofold: (i) to assess the feasibility of measuring Tc using the CorTemp^®^ system in a group of individuals of varying body weight and adiposity under free-living conditions, as well as under standardized laboratory conditions with concomitant EE measurements and (ii) to determine the accuracy of Tc capsules of the CorTemp^®^ system compared with gold standard mercury (Hg) thermometers.

## Materials and Methods

### Human Study

#### Subjects

A total of 27 subjects (9 men and 18 women), aged (mean ± SD) 25 ± 6 years, were recruited from university advertisements (*n* = 17; lean subjects) or from a weight management clinic (*n* = 10; overweight or obese). Lean participants had a BMI (mean ± SD) of 21 ± 2.4 kg/m^2^ while overweight/obese subjects had a BMI (mean ± SD) of 33.3 ± 3.7 kg/m^2^. None of the subjects had any diseases or were taking any medication affecting EE. Exclusion criteria included individuals who had known or suspected obstructive disease of the gastrointestinal (GI) tract, disordered gag reflex, previous GI surgery, felinization of the esophagus, subjects who might undergo MRI scanning during the period the ingested capsule was within the body, subjects with hypomotility disorders of the GI tract, subjects with a cardiac pacemaker or other implanted electromedical devices; these criteria were assessed using a questionnaire administered as part of an anamnesis interview with a medical doctor. For standardized laboratory measurements of EE, all participants were tested under conditions of overnight fasting (~12 h after the last meal) and having abstained from alcohol, smoking and caffeine, as well as moderate or vigorous exercise for 24 h before the tests. Females were measured in the follicular phase of the menstrual cycle. The study was conducted according to the guidelines laid down in the Declaration of Helsinki and all procedures involving human subjects were approved by the Commission Cantonale D’éthique de la Recherche sur L’être Humain, the ethics committee for human research in the Swiss cantons of Vaud, Fribourg, Neuchâtel, and Valais. Written informed consent was obtained from all subjects prior to participation in the study.

#### Core Body Temperature

Core body temperature was monitored using ingestible capsules (CorTemp^®^, HQ Inc., Palmetto, FL, USA): weight 2.75 g, length 23 mm, and diameter 10.25 mm. As the capsule transits through the GI tract, it transmits the temperature measurement values every 20 s *via* a radio signal to an external data recorder worn around the volunteer’s waist. Manufacturers’ instructions were followed for administration of the ingestible capsule and no initial calibration was performed, as per manufacturers’ recommendations. However, the temperature reactivity of all capsules was tested prior to ingestion by comparing them to an Hg thermometer in water at room temperature and subsequent warming of the water. If no change in temperature was observed in response to warmed water, then the capsule was discarded and another used.

#### Protocol

**Figure 1:**
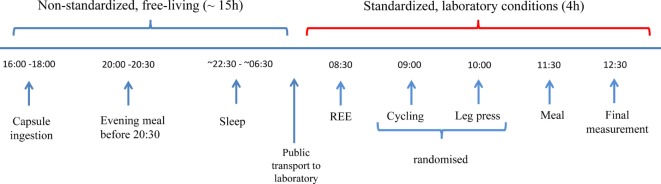
Study design. Capsules were ingested prior to 18:00 the night before the experiment. Subjects were instructed to take their evening meal no later than 12 h before their scheduled test the following morning to ensure all subjects were fasted for ~12 h. Subjects were given instructions on when to go to bed and to wake up the next morning and were advised to take motorized/public transport to the laboratory. Following a rest period, baseline resting energy expenditure (REE) measurements were taken for 30 min, followed by a series of low-intensity exercise tests, and followed by a 600 kcal mixed meal.

An outline of the entire study protocol is illustrated in Figure 1. All subjects ingested the capsules with a glass of water between 16:00 and 18:00 the evening prior to the test. Following ingestion, the temperature-recording device was fitted to a belt worn around the subject’s waist. Subjects were instructed to remove the recorder when bathing, but otherwise not to remove it, even during sleep. Subjects were provided with an activity log and asked to record the timing of their movements, meal consumption, as well as sleep and awakening times (advised to be around 22:30 and 06:30, respectively). They were also requested to note the time corresponding to any loss of temperature signal from the recording device particularly after a defecation event; this being indicative of the time taken for evacuation of the capsule from the body after its ingestion and referred to as expulsion time. Subjects were instructed to consume their evening meal not later than 20:30 and the standardized EE measurements were undertaken after an overnight fast of ~12 h. All tests started the next morning between 08:30 and 09:00 and were performed in an air-conditioned (23°C) quiet laboratory, with subjects at thermal comfort. Subjects were driven by car to the laboratory or took public transport. Each Tc recorder was checked upon arrival to ensure the capsule was still in the GI tract. Subjects were asked to empty their bladders if necessary and to sit in a comfortable armchair at thermal comfort. They were instructed to wear light clothing consisting of a t-shirt, shorts, and sports shoes. Subjects were asked to relax and avoid unnecessary movement, and in order to reduce boredom and accompanying stress, they were permitted to watch a calm movie or a documentary throughout the metabolic measurements. EE was measured using indirect calorimetry (Quark CPET, COSMED, Albano Laziale, Italy). Heart rate was monitored using an adjustable belt (COSMED). Baseline readings were taken for at least 30 min, after which subjects performed incremental low power cycling across 10–50 W at 60 rpm (17) and low-intensity isometric (leg press) exercise (18); these two low-level exercise tests being performed in a randomized order. Regardless of the order of the exercise tests, all experiments were followed by a 600 kcal meal consisting of two slices of toast bread (Migros, Switzerland), 25 g jam (Héro, Nestle, Switzerland), 15 g butter (Migros), and a 300 ml nutritional supplement (Resource Energy, Nestle). Metabolic measurements continued for a further 60-min postmeal. This postprandial timing was chosen on the basis of a previous (unpublished) study in our laboratory where the increase in EE after this mixed meal of 600 kcal was found to reach a plateau in about 30 min, with this steady state postprandial EE being maintained for about an hour; i.e., between 30 and 90 min postmeal.

### *Ex Vivo* Validation Study

The ingestible capsule temperature sensors were validated for accuracy against two Hg thermometers (range 34–42°C; VWR, Dietikon, Switzerland). These two thermometers were compared against a third Hg thermometer with a larger temperature range (0–100°C), which was calibrated using ice (0°C) before reading. Each ingestible capsule was fixed in place in a digital water bath (2.6 L; VWR, Dietikon, Switzerland) directly adjacent to the two Hg thermometers, as well as two electronic EBI310 thermometers with TPX220 probes (EBRO, Ingolstadt, Germany). All equipment were placed in the water bath prior to stepwise heating and during the gradual increase in temperature from room temperature to a starting temperature of 35°C. Based on previous papers reporting submersion times in the range of 4–5 min with multiple submersion temperatures (19, 20), a 0.5°C stepwise increase in temperature was performed from 35 to 40°C with a period of stabilization of 8 min after each 1°C increase in temperature. Recordings were taken during the stabilization period, every minute for 8 min. Ingestible capsules were deemed to be stable after 5 min based on a difference between measurements of ≤0.1°C. Once a stabilization period had been identified, capsules were then compared against the Hg and electronic thermometers using the same stepwise increase in water bath temperature from 35 to 40°C; this test for accuracy being performed in the morning and afternoon for 2 or 3 days. Investigations were carried out using only two capsules at a time with one recorder per capsule. The CorTemp^®^ recording device was placed within 20 cm of the capsule. Signal interference from other equipment was minimized by turning off all computers and other devices in the room where the water bath was located. The stepwise increase in temperature was performed in two sets of capsules: the first set was measured at three time points (0, 17, and 24 h) and the second set measured at six time points (twice per day, morning, and afternoon) for three consecutive days. All investigations were carried out by the same investigator.

### Data Analysis

Temperature values for both studies were downloaded from the CorTemp^®^ recording device. For the *ex vivo* validation study, in addition to the computer download, data were also manually recorded from the device in real-time. Outlier data points were excluded manually if they were greater than 2SD from the mean of the nearest six values (three preceding and three proceeding) not including the outlier value. During the *in vivo* study, the mean temperature and SD were calculated over the following time intervals for each subject: in bed before and during sleep, soon after arousal, as well as under laboratory conditions, namely during resting EE measurement (30 min), ergometry cycling (30 min), intermittent isometric leg press (90 min), and postprandial period (60 min). Valid Tc data were available for a subset of 21 subjects during EE measurement and during sleep for 24 subjects.

### Statistical Analysis

Data on changes in EE or Tc across time in response to exercise or meal were assessed by ANOVA with repeated measures. For the *ex vivo* validation studies, difference scores and the level of agreement were calculated for each comparison of temperatures: (i) capsule vs. Hg, (ii) electronic vs. Hg, and (iii) capsule vs. electronic. The mean difference in temperature was calculated to determine systematic bias between the devices. This was done for all days of testing for each capsule. Bland–Altman plots were generated to compare the difference score to the mean of the two of the three devices (capsule, Hg, and electronic). Linear regression was used to determine the relationship between the temperature readings of the capsules and the Hg or electronic thermometers. Data were analyzed using Statistix software (version 8; Tallahassee, FL, USA) and are presented as mean ± SD. Statistical significance was set at the level of *p* < 0.05.

## Results

### Expulsion Time of Capsules from the Body

**Figure 2:**
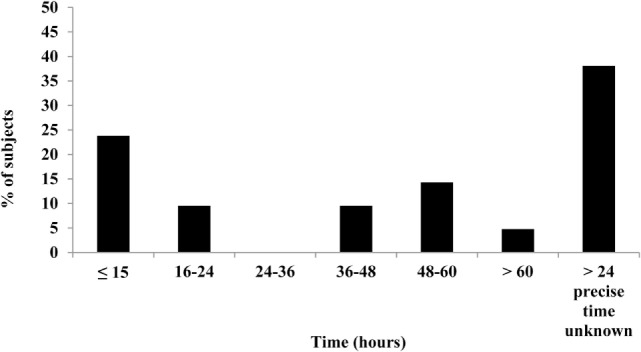
Expulsion times of capsules after ingestion. Complete data available for 21 (out of 24) subjects. Bar chart illustrates the percentage of subjects who expelled the Tc capsule over a period of 13–82 h. Beyond the experimental time period in the laboratory, the precise time of capsule evacuation was unknown for obese subjects (*n* = 8) and is indicated by the last bar in the chart.

The time for the capsules to pass through the GI tract was found to be 31 h on average (range 13–82 h), with evacuation time of ≤15 h in five subjects (24%; Figure 2). Of these five subjects, three were female; mean age was 24 years and mean BMI 23 kg/m^2^ (three normal weight, one overweight, and one obese). Based on gender, mean ± SD expulsion times of 27 ± 15 and 36 ± 24 h were observed for men and women, respectively; no significant between-gender differences were detected.

### Nocturnal Decline in Tc

**Figure 3:**
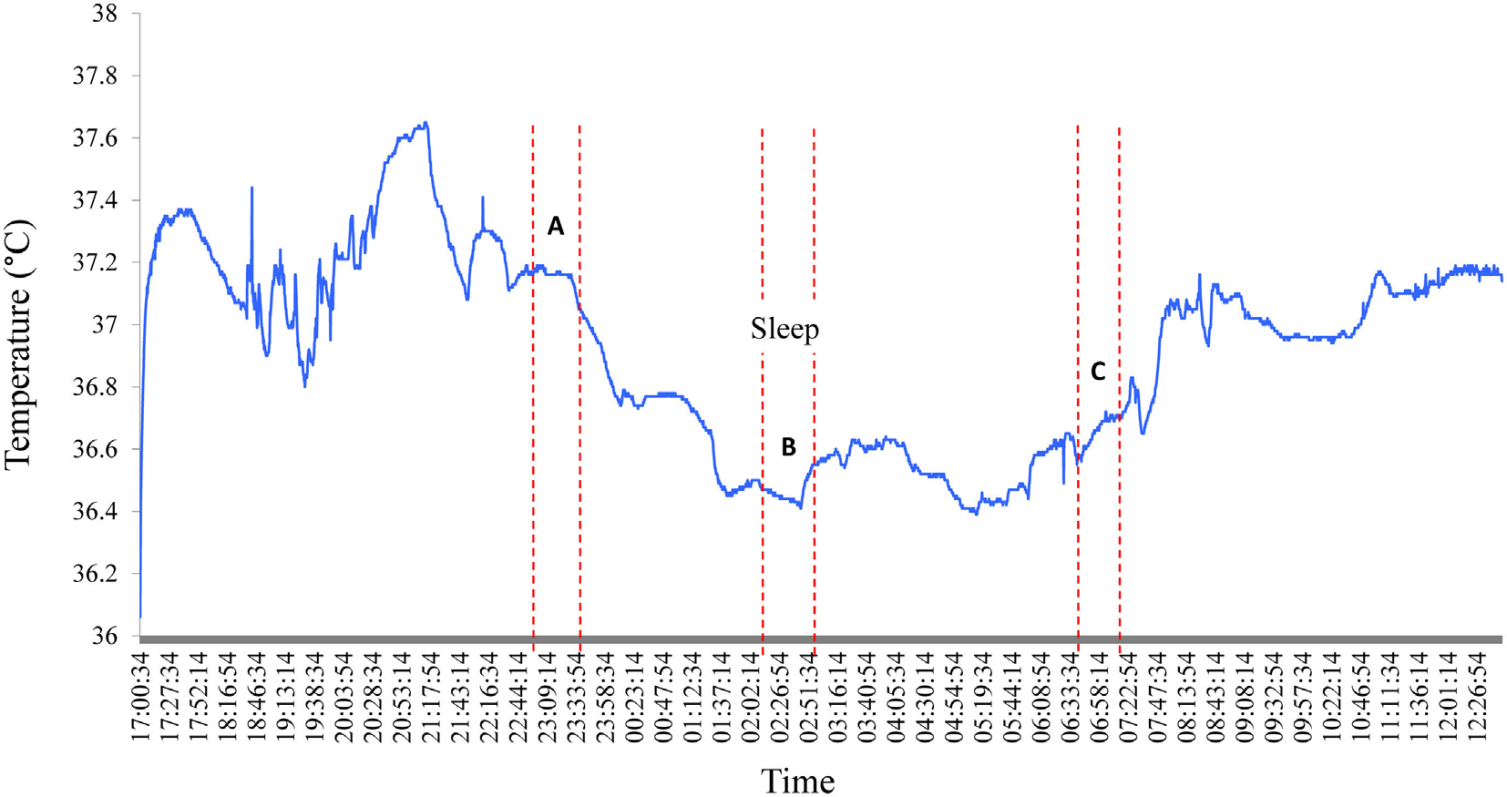
Nocturnal changes in core body temperature in one subject. A typical Tc profile for one subject shows the nycthemeral and nocturnal decline in Tc. A, B, and C correspond the following time periods: **(A)** the highest 30-min average value before sleep onset, **(B)** the lowest 30-min average value during sleep, and **(C)** the first 30-min average value after arousal.

A decline in night time Tc was observed for all subjects in the current study; a typical sleep pattern for one subject is shown in Figure 3. Furthermore, the large range in Tc obtained over the awake or sleep periods, and corresponding to temperature oscillations of 0.4°C, may in part be explained by a change in total heat production not necessarily in phase with the change in total heat loss. In further analysis of individual data while lying in bed, Tc was assessed at the following time points: (A) the highest 30-min average value before sleep onset, (B) the lowest 30-min average value during sleep, and (C) the first 30-min average value after arousal. These three values and the differences between them (B − A; B − C; C − A), computed for each subject, are presented in Table 1. The average drop in Tc during sleep (B − A) was −0.6 ± 0.1°C. The average difference between the maximum drop in Tc and postarousal (B − C) was −0.4 ± 0.1°C. Overall, the change in Tc from before sleep onset to postarousal (C − A) was −0.2 ± 0.1°C.

**Table 1:** Core body temperature (°C) in bed before (A) and during (B) sleep, and after arousal (C).

Subject#	Gender	Body mass index (kg/m^2^)	A (before sleep)	B (during sleep)	C (after arousal)	B − A	B − C	C − A
1	F	17.9	37.2	36.5	36.8	−0.7	−0.4	−0.4
2	M	25.9	36.8	36.2	36.9	−0.5	−0.6	0.1
3	F	21.8	37.2	37.0	37.0	−0.2	0.0	−0.2
4	M	23.6	36.7	36.2	36.7	−0.5	−0.5	0.0
5	F	20.0	37.3	37.1	37.5	−0.1	−0.3	0.2
6	F	18.8	37.3	36.8	37.2	−0.4	−0.4	−0.1
7	F	17.6	37.3	36.9	37.0	−0.4	−0.1	−0.3
8	F	20.9	37.4	37.0	37.2	−0.3	−0.2	−0.1
9	M	24.5	36.8	36.5	36.7	−0.3	−0.2	−0.1
10	F	18.1	37.0	36.4	36.9	−0.6	−0.5	−0.1
11	M	19.8	37.1	36.5	36.6	−0.7	−0.1	−0.6
12	M	20.1	37.1	36.3	36.4	−0.8	0.0	−0.7
13	M	23.8	37.2	36.4	36.6	−0.7	−0.2	−0.5
14	M	21.4	36.7	36.2	36.4	−0.4	−0.1	−0.3
15	F	30.0	36.8	36.4	36.7	−0.4	−0.3	−0.1
16	M	37.5	36.4	36.0	37.3	−0.5	−1.4	0.9
17	F	33.0	37.1	36.5	37.7	−0.6	−1.3	0.6
18	F	29.0	37.1	36.4	36.6	−0.6	−0.2	−0.4
19	F	23.2	37.6	36.1	36.5	−1.5	−0.3	−1.2
20	F	36.9	37.5	37.1	37.6	−0.5	−0.5	0.0
21	F	22.2	37.0	35.9	36.7	−1.1	−0.8	−0.3
22	F	30.8	37.7	36.9	37.0	−0.8	−0.1	−0.7
23	F	30.0	37.7	36.9	37.3	−0.9	−0.4	−0.4
24	F	39.6	37.0	36.5	37.2	−0.5	−0.7	0.2
**Mean**			**37.1**	**36.5**	**36.9**	**−0.6**	**−0.4**	**−0.2**
**SD**			**0.3**	**0.4**	**0.4**	**0.3**	**0.4**	**0.4**
*Pair-wise comparisons*						*p < 0.001*	*p < 0.001*	*p < 0.05*

*Values represent the mean of 30-min temperature readings (1,800 individual values) for subjects with complete sleep data (*n* = 24). Temperature was measured at the following time points: (A) the highest 30-min average value before sleep onset, (B) the lowest 30-min average value during sleep, and (C) the first 30-min average value after arousal. Sleep and awakening times were self-reported by each subject in an activity log book. Differences between time points (A − B; A − C; and B − C) were calculated using paired *t*-test*.

*F, female; M, male*.

### Effect of Low-Intensity Exercise and a Mixed Meal on Tc and EE

**Figure 4:**
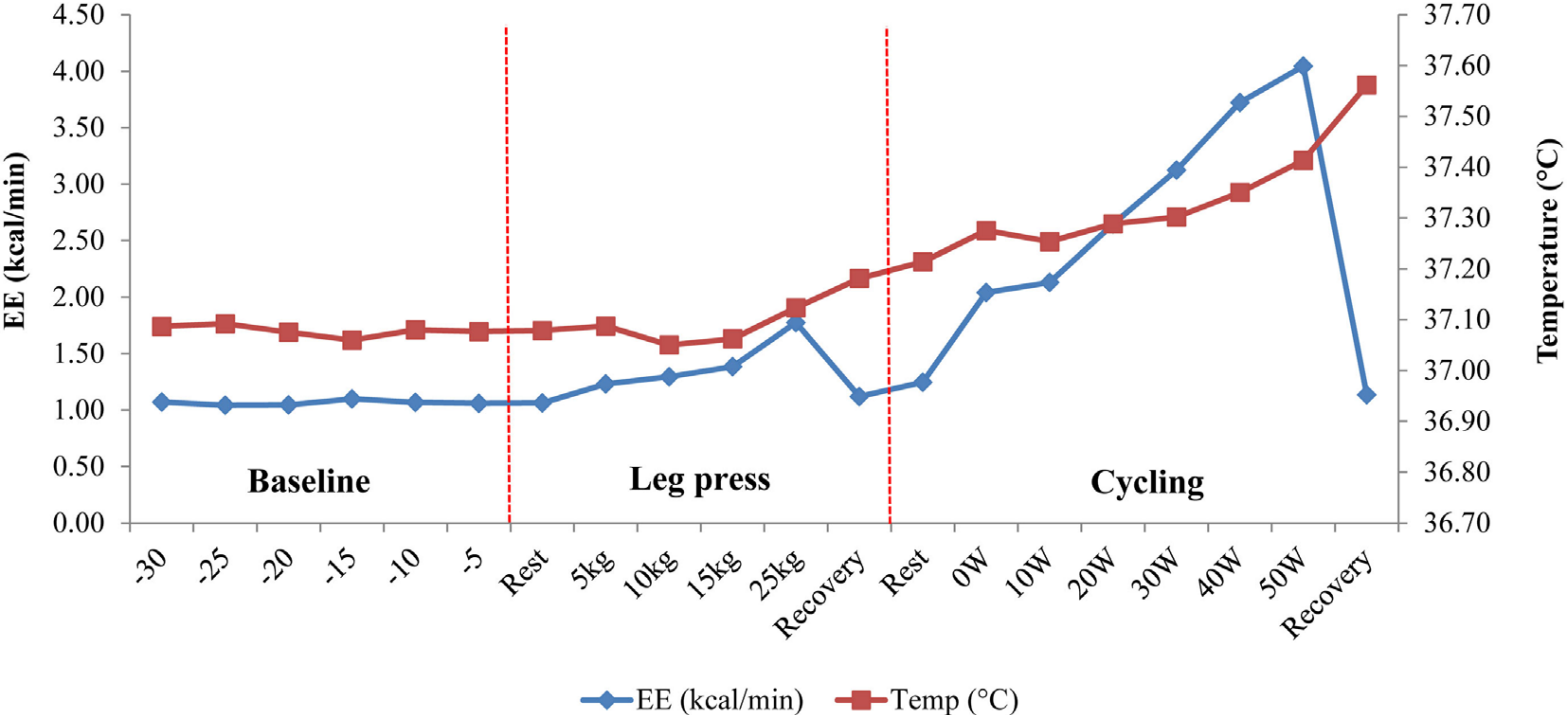
Temperature change in response to dynamic (cycling) and isometric (leg press) exercises in one subject. Following a 30-min baseline measurement period, energy expenditure (EE) and core body temperature (Tc) were measured simultaneously during an isometric leg press exercise (5–25 kg) followed by low power cycling (0–50 W) on an ergometer bicycle. Note the sudden drop in EE during “Recovery” periods, which contrasts with the continuous increase in Tc, probably due to thermal lag time between total heat production and total heat loss (3).

Mean changes in Tc and EE in response to the exercise tests and to ingestion of a mixed meal (600 kcal) are shown in Table 2. Dynamic exercise consisting of low-power cycling resulted in a small increase in Tc but only as from 40 W (+0.1°C) reaching statistical significance at 50 W (+0.2°C, *p* < 0.05) compared to resting precycle values; the EE at 40 and 50 W being ~3–4 times higher than at rest (i.e., ~3–4 METs). No significant differences were observed for Tc in response to intermittent leg press exercise resulting in EE < 2 METs or to a 600 kcal mixed meal where the peak increases in postprandial EE was <25% relative to premeal (fasting) values. Furthermore, no significant differences in Tc in response to the light exercise or meal were observed due to differences in BMI. An example of Tc profile showing changes in response to dynamic and isometric exercise is presented in Figure 4.

**Table 2:** Low-level structured exercise and meal-induced changes in energy expenditure (EE) and core body temperature (Tc).

	Cycling *(dynamic)*			Leg press *(isometric)*			Mixed meal *(at rest)*		
	Rest	50 W	Δ	Rest	25 kg	Δ	Pre	Post	Δ
*n*	*27*	*27*		*25*	*25*		*18*	*18*	
EE (kcal/min)	1.17 ± 0.22	4.78 ± 0.99	3.61 ± 0.94***	1.10 ± 0.26	1.65 ± 0.36	0.55 ± 0.17**	1.12 ± 0.22	1.23 ± 0.22	0.11 ± 0.14**
*n*	*21*	*21*		*20*	*20*		*19*	*19*	
Tc (°C)	37.1 ± 0.23	37.3 ± 0.23	0.17 ± 0.12***	37.2 ± 0.27	37.1 ± 0.27	−0.08 ± 0.18*NS*	37.3 ± 0.30	37.2 ± 0.23	−0.05 ± 0.22*NS*

*Values are mean ± SD*.

*EE, energy expenditure; Tc, core body temperature*.

Statistically significant by paired t-test: **p < 0.01; ***p < 0.001; NS, not significant.

### *Ex Vivo* Validation of Capsule Accuracy

#### Response Time

**Figure 5:**
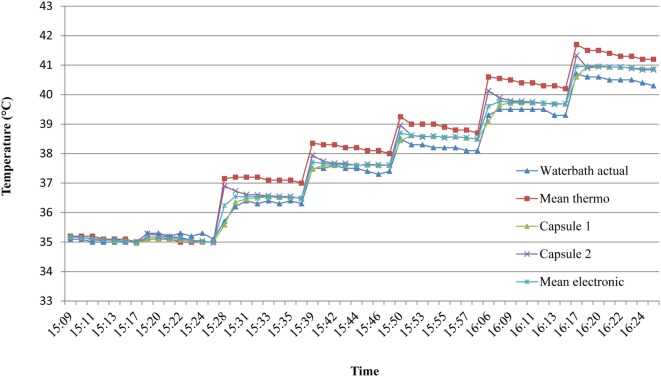
Time to reach temperature stability in *ex vivo* studies. To assess the time required for the capsules to equilibrate with water temperature, consecutive 60 s stepwise changes in temperature in the range 35–40°C were plotted over 8 min. A consistent change of less than 0.1°C from one reading to the next indicated thermal equilibrium, which was deemed to be by the fifth minute. Mean thermo = mean of two mercury thermometers. Mean electronic = mean of two identical electronic (EBRO) thermometers.

A stability test was first carried out to determine the time required for the capsule temperature to equilibrate following a step-change in water bath temperature. Consecutive 60 s changes in temperature in the range 35–40°C were plotted over 8 min periods (Figure 5) and a consistent change of <0.1°C from one reading to the next was taken to indicate equilibration; this was found to be reached within 5 min. Therefore, all further readings were taken after 5 min of equilibration at a given water temperature.

#### Mercury Thermometer Readings

**Figure 6:**
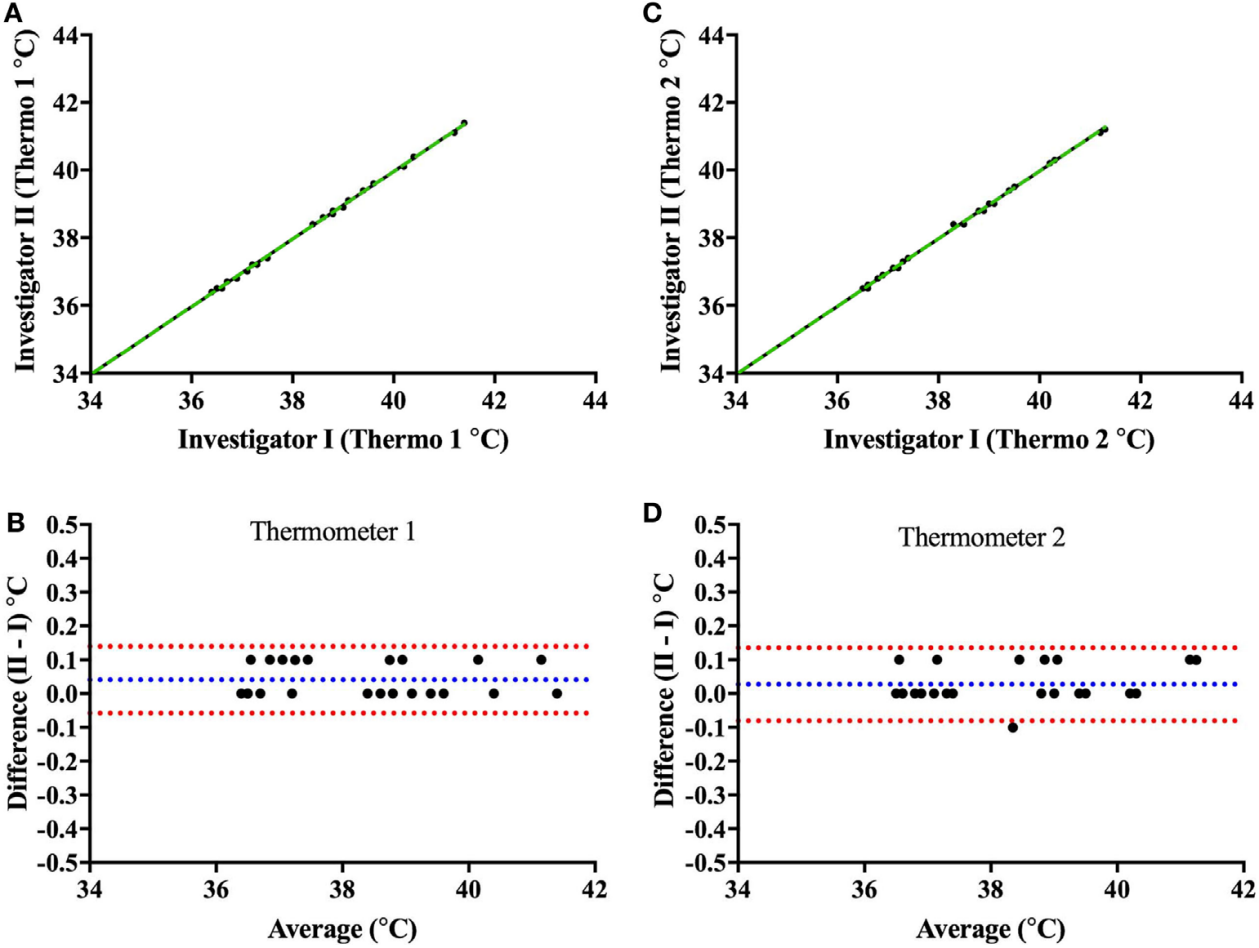
Interinvestigator-associated bias for each Hg thermometer readings. Top panels show plots comparing readings from both investigators (I vs. II) for Hg thermometer 1 **(A)** and Hg thermometer 2 **(C)** across the range of 35–40°C; the dashed green lines being the lines of identity. To note that the two investigators I and II (authors CM and JC, respectively) read the thermometer temperature within a few seconds of each other—and values recorded blinded from each other. Bottom panels show Bland–Altman plots of the difference vs. the average of readings comparing investigators I and II for Hg thermometer 1 **(B)** and Hg thermometer 2 **(D)**. Blue and red dashed lines indicate the mean bias and limits of agreement, respectively.

**Figure 7:**
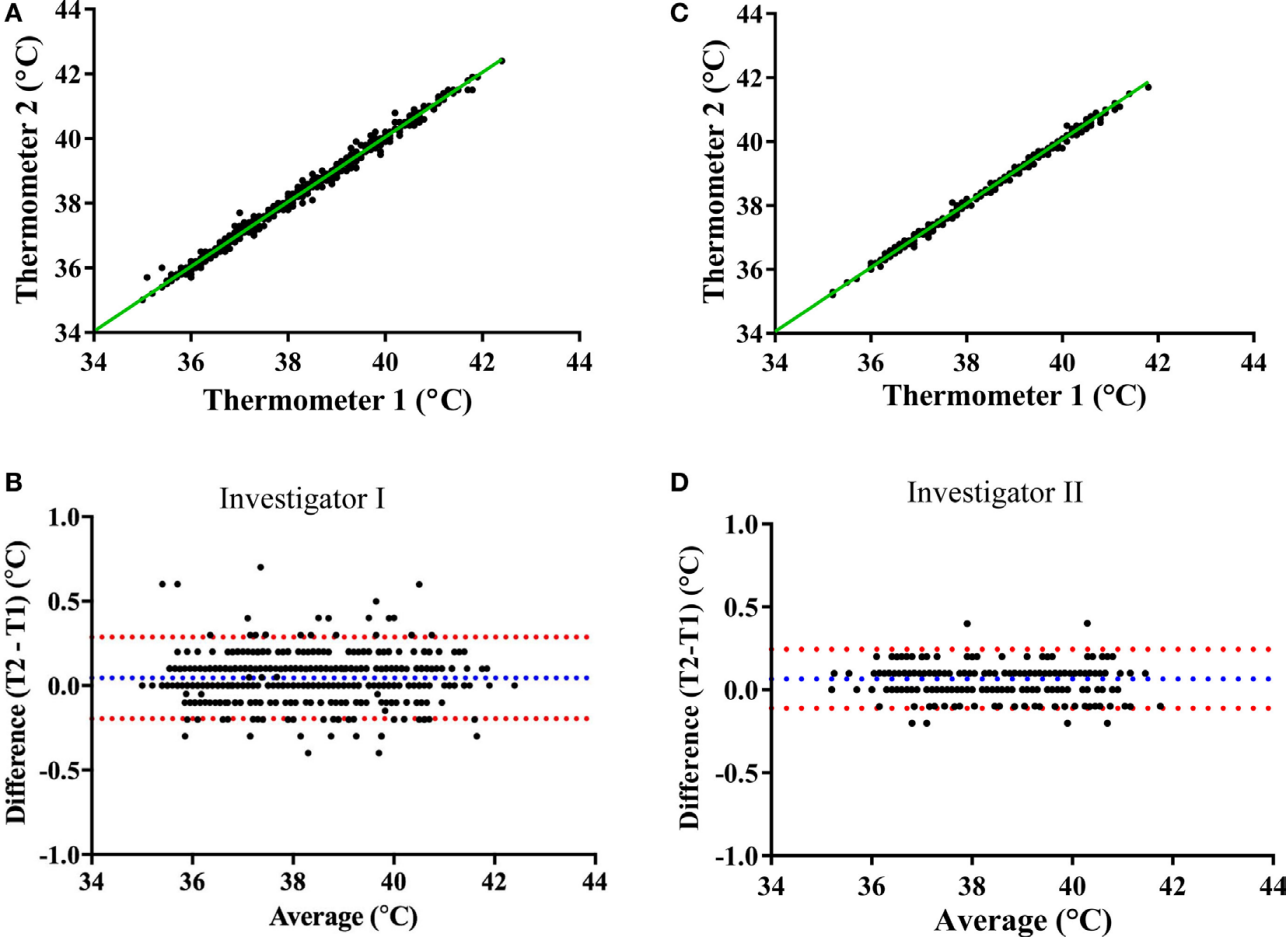
Mercury thermometer bias as measured by each investigator on separate occasions. The top panels compare the temperature readings from the Hg thermometers 1 and 2 (Thermo 1 vs. Thermo 2) across the range of 35–40°C by investigator I **(A)** or by investigator II **(C)**; the dashed green lines being the lines of identity. The bottom panels show Bland–Altman plots of the difference vs. average of readings from the two Hg thermometers (T1 and T2) made by investigator I **(B)** or investigator II **(D)**; blue and red dashed lines indicate the mean bias and limits of agreement, respectively.

Prior to validating the capsule temperature against the two Hg thermometers, a blinded test was carried out between two independent investigators I and II to determine the potential influence of investigator (parallax) error in reading the temperature from the Hg thermometer in the range of 35–40°C. The mean interinvestigator bias was 0.041°C (limits of agreement: −0.06 to 0.14°C) for the Hg thermometer 1 (Figures 6A,B) and 0.027°C (limits of agreement: −0.08 to 0.14°C) for thermometer 2 (Figures 6C,D). Furthermore, the average difference (bias) between the two Hg thermometer readings (across 35–40°C calibration) when determined by each investigator on separate occasions (a few months apart) was found to be 0.045°C (limits of agreement: −0.196 to 0.287) for investigator I (Figures 7A,B), and 0.066°C (limits of agreement: −0.111 and 0.244) for investigator II (Figures 7C,D). There were no significant differences between investigators for thermometer 1 (*p* = 0.932) or thermometer 2 (*p* = 0.953), therefore, all subsequent measures of agreement between the two thermometers were carried out by one investigator using a 0.5°C stepwise increase in temperature across the range 35–40°C.

#### Comparison of Ingestible Capsule vs. Mercury Thermometers

**Figure 8:**
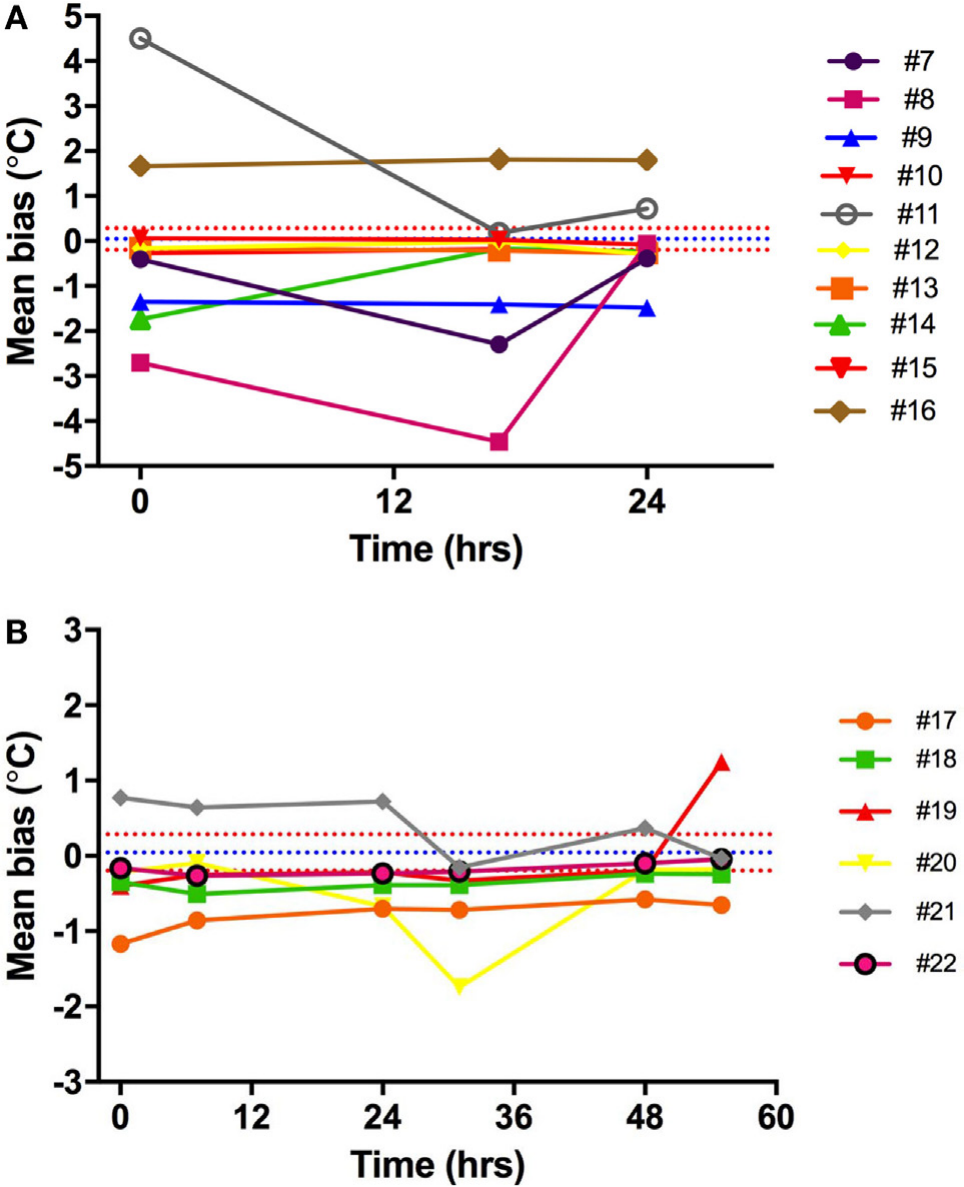
Capsule vs. Hg thermometer bias. Mean bias for temperature reading of each capsule vs. Hg thermometer at different time points over 24 h **(A)** or 55 h **(B)**. Different colored lines represent different capsules. Blue and red dashed lines are the mean bias and limits of agreement, respectively, indicating the potential variability of investigator (parallax error) for reading the Hg thermometer.

**Figure 9:**
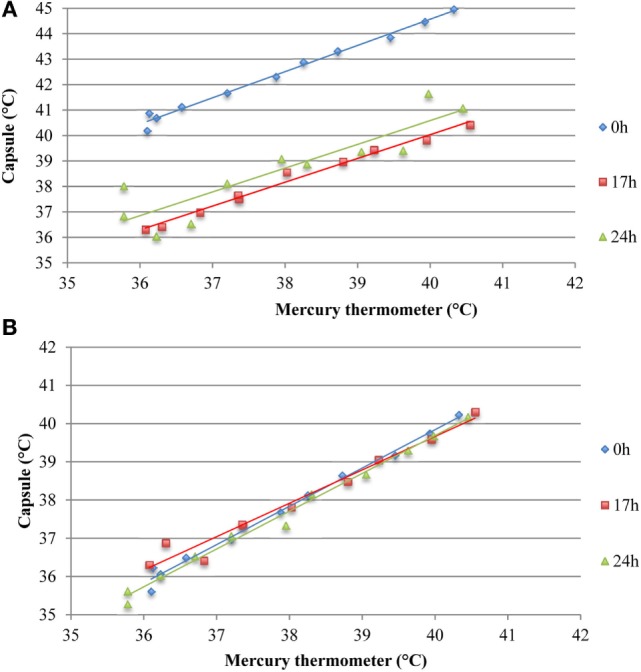
Assessing potential temperature drift over 24 h observed in two capsules measured simultaneously *ex vivo*. **(A)** Capsule 11 shows an initial bias at 0 h, which is not present at 17 or 24 h. **(B)** Temperature readings from capsule 12 were comparable to the mean of two mercury thermometers over 24 h, thereby indicating absence of drift.

Ingestible capsule temperature was compared with the mean of the two Hg thermometers in the range of 35–40°C at time points within 2 or 3 days. Examination of the initial mean bias between capsules vs. Hg thermometer indicates that ~50% of capsules yielded temperature readings that were well outside the limits of the investigator’s reading (parallax) error (within ±0.1°C) assessed above; the initial bias for these capsules exceeded ±0.5°C, as shown at time point 0 in Figure 8. Furthermore, the bias between each ingestible capsule and the Hg thermometer reading was not consistent over time for all capsules; it changed substantially in one out of every four capsules. As shown in Figure 8A, there was a drop of 4.5°C for capsule 11 by 17 h after the initial calibration, an increase of ~2°C for capsule 14 by 17 h, and a drop of ~1.5°C by 17 h followed by an increase of 4.5°C by 24 h for capsule 8. Similarly, as shown in Figure 8B, capsule 20 showed a drift of about −1°C by 24 h, followed by an additional −1°C drop 12 h later, and returned to initial value by 48 h, while capsule 21 showed a drift by −1°C by 24 h, followed by a transient 0.5°C drift by 48 and 55 h. An example of the drift in temperature calibration can be seen in Figures 9A,B for one capsule out of two measured at the same time (capsules 11 and 12). Capsules and Hg thermometers were compared over the range 35–40°C at 0.5°C intervals over 24 h. Readings for capsule 12 were comparable over the three time points of measurement, whereas capsule 11 had an initial bias of 4.5°C compared with the Hg thermometers, but by 17 h, readings were comparable.

#### Comparison of Electronic vs. Mercury Thermometers

**Figure 10:**
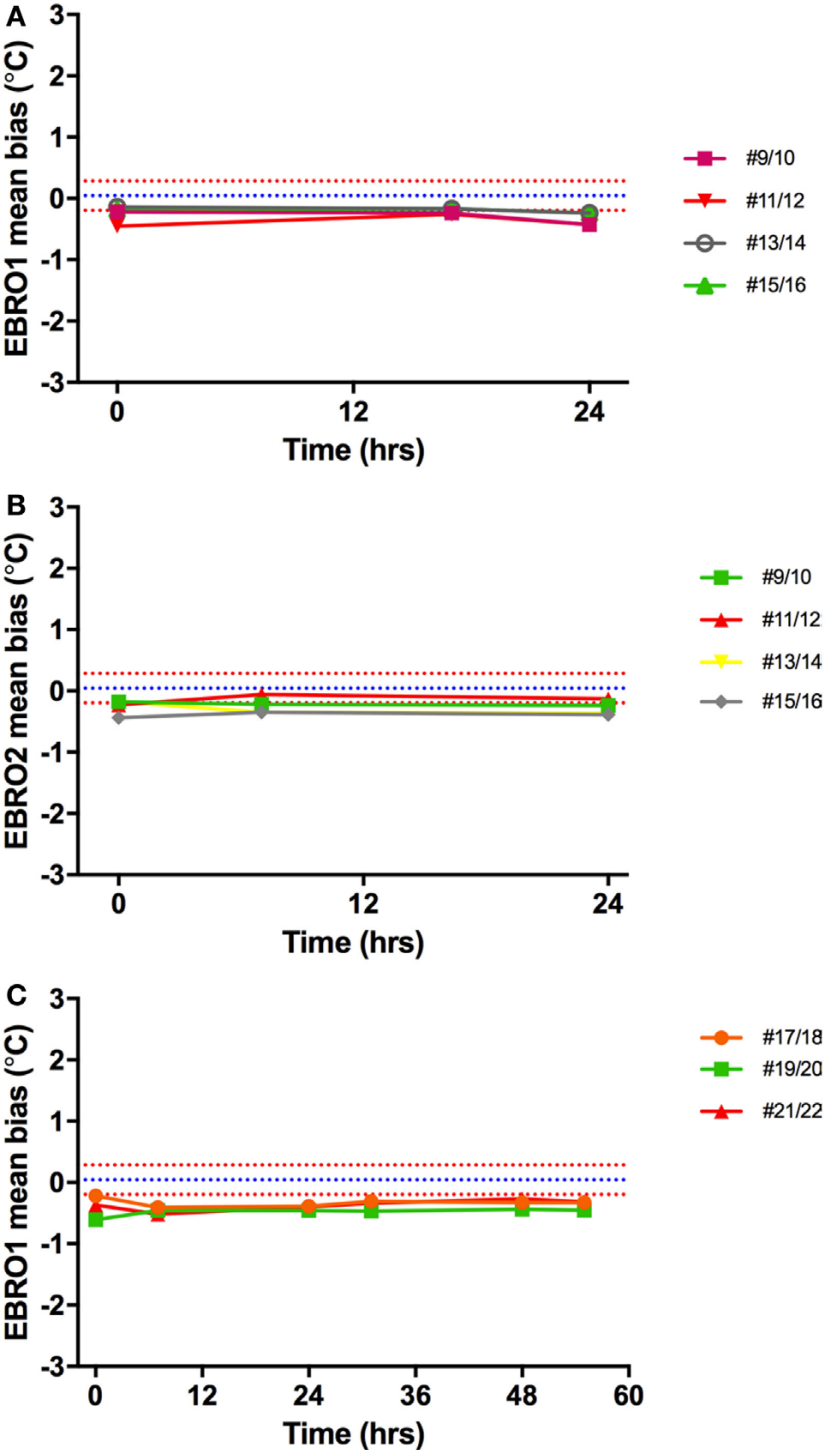
Temperature bias of electronic thermometers vs. Hg thermometers. The mean difference between Hg thermometers and EBRO1 measured over 24 h **(A)** and 55 h **(C)**, and EBRO2 [24 h; **(B)**] is shown. Different colored lines represent measurements made on different days simultaneously with core body temperature capsules. Blue and red dashed lines are the mean bias and limits of agreement, respectively, a narrow window of uncertainty due to the potential variability of investigator (parallax error) for reading the Hg thermometer.

Validation of the two identical electronic thermometers (EBRO1; EBRO2) against the Hg thermometers across 35–40°C (concomitant to validation of capsules) showed a mean bias of −0.41°C for electronic thermometer 1 and −0.42°C for electronic thermometer 2. There was no drift in electronic temperature readings vs. Hg thermometer over time (Figure 10).

#### Comparison of Ingestible Capsule vs. Electronic Thermometer

In an additional *ex vivo* validation study, 28 ingestible capsules were compared against one or both of these electronic thermometers on a continuous basis over 2 or 3 days with readings recorded every 20 s. Drift in capsule temperature exceeding ±0.5°C was found to occur within 24 h in eight capsules and to occur between 24–48 h in one capsule (Table 3).

**Table 3:** *Ex vivo* non-consistent bias (drift) over first and second day in comparing 28 ingestible capsules vs. one or two identical electronic thermometers (EBRO1 and EBRO2); values exceeding ±0.5°C are shown in bold.

Date	# capsules	EBRO1	EBRO2	EBRO1	EBRO2
	
		0–24 h bias		24–48 h bias	
29.10.2015	1	0.4	–	0.4	–
05.11.2015	1	0.4	–	0.4	–
23.11.2015	1	0.2	0	0.2	0.0
27.11.2015	1	0.3	0.5	–	–
04.12.2015	1	0.2	–	0	–
	2	0.2	–	0	–
15.12.2015	1	0.1	0.3	–	–
17.12.2015	1	−0.1	**0.6**	–	–
18.12.2015	1	0.2	0.4	0.2	0.1
	2	−0.2	0	0.1	0
23.12.2015	1	−**0.6**	−0.1	−0.3	0
	2	0.4	**0.9**	0.2	0.5
10.02.2016	1	0.3	0.1	–	–
	2	0.2	0	–	–
14.03.2016	1	0.2	**2.2**	–	–
	2	0	**1.9**	–	–
30.05.2016	1	−**0.6**	−0.1	–	–
07.06.2016	1	0.2	0.2	–	–
	2	0.2	−0.5	–	–
13.06.2016	1	**1.6**	–	**0.6**	–
	2	−**0.7**	–	0.4	–

## Discussion

The aims of this study were to investigate the feasibility of measuring Tc using the CorTemp^®^ system in a group of healthy subjects across a wide range of BMI and to validate the accuracy of these temperature capsules *ex vivo*. The main findings were (i) a nocturnal drop in Tc among all subjects, (ii) a modest increase in Tc in response to low-level dynamic cycling exercise exceeding 40 W (>3 METS), but no change in Tc in response to low-level isometric exercise (<2 METs) or to a 600 kcal meal, and (iii) the results of the *ex vivo* validation study revealed a substantial mean bias (exceeding ±0.5°C) between the Tc capsule readings and Hg thermometer (as well as against electronic thermometers) in half of the capsules, and furthermore when examined over 2 or 3 days, the initial bias (small or large) drifted importantly in one out of every four capsules. Thus, part of the variability observed *in vivo* could stem from the drift factor.

The interest in Tc as a potential thrifty metabolic trait stems from a few studies, which have shown a relationship between Tc and BMI or fat mass (10–13). However, in other studies no association was found (14) or no differences were detected between lean and obese (6). Given that Tc changes in the range of 0.4°C are reported in these studies, it is important that the device used to measure Tc should be accurate and precise enough to detect such small changes. In the process of measuring continuous Tc in subjects as part of this study, we identified a number of expected and unexpected findings, which may influence the accuracy of Tc measurement in humans and may be of interest to researchers in the field. These findings are discussed below in more detail.

### Nocturnal Decline in Tc

Homeotherms conserve energy by lowering body temperature; an example of this can be seen at night when body temperature declines during sleep, part of which is explained by the progressive disappearance of the residual postprandial thermogenesis of the evening meal which often lasts 5–7 h. As expected, a decrease in Tc was observed among all subjects in the current study, which confirms previous reports of the general population (6, 21, 22). Greater nocturnal declines in Tc (up to 2°C) have been reported in nomadic populations such as the Australian aborigines (23) and nomadic Lapps (24) and have been suggested to represent an energy-saving adaptation. In the current study, the average decline in Tc from sleep onset to the lowest night time temperature was −0.6°C (range −1.49 to −0.1). This signature drop in Tc provides an interesting point of comparison in Tc studies; however, measurement of body movement during sleep should be undertaken to allow researchers to account for individual variations in movement during sleep, which may affect Tc readings.

### Capsule Expulsion Time

Internal Tc is measured as the telemetric capsule moves through the GI tract. Therefore, the speed of progression of the capsule through the gut will have a significant influence on the time taken for evacuation of the capsule from the body (i.e., expulsion time) and hence on the duration of Tc recording. The average expulsion time in the current study was 31 h, with a large variation among subjects (range: 13–82 h). In those in whom the capsule was evacuated in less than 15 h (*n* = 5), there were no apparent differences in gender (three females and two males) or in BMI category (three normal and two overweight/obese). Similar expulsion time is reported in the literature, with a range of between 8 h (25) and 5.6 days (26), and is likely to be influenced by the transit time of food through the gut. Such food transit time has been shown to vary between individuals according to the type and volume of food/fluid ingested. Additional factors reported to affect transit time in the general population include alcohol (26), heavy exercise (27), age (older adults have greater transit time) (28), and gender (females have a greater transit time than males) (29). In the current study, subjects were advised to avoid exercise and alcohol for 24 h prior to the test to reduce the effects on transit time and EE. Dietary fiber, particularly wheat bran, has also been shown to decrease colonic and whole gut transit time (30)—an effect that is likely to be exacerbated if it is not associated with an increase in water drinking. In the study by Song et al. (29), females had a significantly longer transit time than males and the authors suggest that female hormones, and in particular progesterone, may be responsible for this difference. In our study, mean expulsion time of the capsule was greater in women than in men (36 vs. 27 h), despite the fact that among those who passed the capsule in ≤15 h, three out of five were women. Although Lee et al. (25) originally recommended ingesting the capsule 6 h prior to data collection to ensure the capsule has reached the intestine, it has since been shown that the location of the capsule in the gut does not substantially affect temperature readings (31). In the current study, capsules were ingested before the evening meal, which has been shown in previous studies to promote transit time (32) and may be one explanation for the rapid expulsion time of the capsule observed in some individuals in the current study. Expulsion time of the capsule is an important factor to consider when conducting studies of this nature to optimize measurement time and minimize data loss.

### Temperature Oscillations

We observed large erratic temperature excursions outside of the physiological range for all subjects, which occurred to a greater frequency in subjects with greater abdominal adiposity (waist circumference > 100 cm). This may relate to noise due to the proximity of the recording device (worn on the waist) to the capsule within the intestine. Greater processing was required for data from these subjects, which reduced the number of data points available. Previous studies have excluded outliers greater than 2SD from the mean (of a particular time period) when the outlier was included in the mean value (6). However, in the current study, such erratic temperature oscillations were often >80°C and if included in the mean would have invalidated the results for that time period. Therefore, manually checking the data for outliers, although time consuming, ensured more representative Tc values. Computer filters can also be used to eliminate the outliers automatically.

### *Ex Vivo* Validation Study

Following these technical difficulties (number and uncertainty of outliers), a validation study was carried out to investigate the accuracy of the capsules measured in the physiological range of 35–40°C, twice per day for 3 days. We identified a substantial bias exceeding ±0.5°C compared to Hg thermometers in half of the capsules, with the bias in some capsules exceeding ±1°C (Figure 8, time point 0). In previously studies, bias of 0.27°C (19) and ±0.4°C (33) have been found, but these values are averages for all capsules studied. Furthermore, the latter study tested capsules at non-physiological temperatures between 40 and 55°C, which may account for some of this difference. Hunt and Stewart (20) have previously shown that the measurement error increased above 41°C. Additionally, Mittal et al. (33) used a Bowman probe instead of an Hg thermometer for comparison, which may also explain some of these differences.

### Investigator-Associated Bias

While considering the source of bias, it is important to note that there is an inherent investigator error in reading the temperature value (parallax error). In the current study, the degree of investigator-associated bias was assessed before comparing the Tc capsules with Hg thermometers. An investigator-associated bias of <0.05°C was identified, and hence of small magnitude. These inherent errors of random nature were taken into account for all subsequent comparisons between capsule and thermometers and constitute a window of uncertainty that should be an important consideration in studies of Tc where minute differences can have a considerable impact on the final result.

### Non-Constant Bias over Time

In the current study, repeated accuracy tests of each capsule recording were taken in order to determine whether or not the accuracy of the capsules changed over time (up to 3 days). This information is vital given that Tc studies generally measure subjects for 24 h or more. We were interested in exploring whether the initial bias observed between the capsules and thermometer, as reported previously (8, 20, 25), changed over time. In 16 additional capsules, which underwent several comparisons against an Hg thermometer and an electronic thermometer over three consecutive days, the bias observed was not consistent over time. Of greater concern was the observation that a drift in bias occurred over time for some capsules (see Figure 9). For example, an initial bias measured at time zero disappeared by 17 h, but in some instances, a new bias of different magnitude was identified at 17 h, which was not present at the initial measurement. This drift in accuracy over time has implications not only for the *in vivo* Tc measurement but also for any calibration procedures undertaken to improve accuracy of the capsules prior to ingestion.

### Calibration

Researchers have sought to remove the bias associated with the Tc capsules by employing an additional calibration step prior to ingestion *in vivo*. However, the literature on capsule calibration prior to Tc measurement is inconsistent. Moreover, the manufacturer of the Tc capsules used in the current study states that additional calibration is not required prior to use. Despite this, a number of validation studies carried out with the same capsule suggest that initial calibration is required as the capsules show a small positive systematic bias compared to Hg thermometers (19, 20). It is recommended that the initial raw values obtained during calibration should be used in regression equations and then corrected for any bias arising between the capsule and Hg thermometer (19, 20). Challis and Kolb (19) found that applying a 5-point correction equation to raw data reduced to 2.2% the number of readings beyond the standard and was superior to using only three data points. More reasonably, the manufacturing company could improve the product in order to eliminate the bias. However, our study highlights a drift in bias over time for some capsules (Figure 9), whereby the initial bias disappeared by 17 h, or new bias occurred over time, which was not present initially. In fact, in our validation studies comparing capsules from three batches of capsules (bought and tested at different times), one in every four capsules showed substantial drift exceeding ±0.5°C in each of the three batches, thereby implying that the initial recommended calibration step might not completely remove bias in the system. Due to inconsistencies or lack of consistent reporting of calibration procedures in the literature, it is difficult to determine the impact of calibration on the results of existing studies. Greater consistency and harmonization with respect to calibration procedures and improved reporting practices are required in future studies.

### Strengths and Limitations

The current study has a number of caveats. First, physical activity was not measured in subjects who underwent Tc measurement prior to the standardized laboratory measurements. The use of accelerometry in future studies would provide activity measurements during the non-standardized component of the experiment, as well as during sleep, which could be incorporated into the analysis and determine the extent to which day-to-day movement influences Tc. Second, the meal subjects consumed the night before the experiment was not strictly standardized. This may have accounted for the differences in expulsion time between subjects and the evolution of Tc during the night. Future studies should consider providing a standardized meal, taking into consideration nutritional factors likely to affect GI transit time, e.g., meal size, dietary fiber content, etc., and which in turn may influence the capsule’s expulsion time. Third, in the measurement of Tc with the ingestible capsule, it is assumed that (i) the anatomical location of the capsule in the gut has very little influence on Tc (e.g., heat production as a result of bacterial fermentation by colon microbiota) and (ii) the presence or absence of food in the gut does not influence Tc significantly. In order to offset this effect of fluctuating Tc over time (Figure 3), the average Tc over the total gastrointestinal transit time should be used. Note that the study was not designed to detect differences in Tc based on gender or BMI. Future studies with larger, equal-sized groups of lean and obese would be useful in addressing some of the inconsistencies in the existing literature on excess body weight and Tc. Strengths of the current study include the fact that Tc and EE were measured simultaneously under standardized conditions among male and female subjects across a BMI range of 20–31 kg/m^2^. This study demonstrates the feasibility of measuring Tc change under free-living and well-standardized laboratory conditions. Temperature changes associated with sleep and physical activity above 3 METs were particularly well identified; for most people, physical activity below 3 METs represents most of the activities performed during the day. Nonetheless, comparison of the ingestible capsule with concomitant rectal temperature monitoring would be desirable in future validation studies, which aim to investigate the sensitivity of the capsules to altered Tc in response to light physical activity or meals. Furthermore, future studies need to address the issue of whether the use of a fixed room temperature might also influence the Tc measurement, particularly when comparing people of different morphology, body fat distribution, and indeed when comparing men and women who may differ in thermal demand (34). Additionally, useful information has been garnered on transit time, which will be important in future studies to ensure a satisfactory measurement period. In this context, the ingestion of the capsule following the 12 h fast may constitute a more standardized approach.

## Conclusion

This validation study is the first to measure a series of Tc capsules for up to 3 days and to identify a change in bias between the capsules and gold standard Hg thermometers, as well as against electronic thermometers. The feasibility of using Tc capsules has been shown, particularly for detecting rapid changes in Tc associated with low-intensity dynamic exercise, but not in response to food or low-intensity isometric exercise; while noting that during daily life, dynamic and isometric activities are intertwined pertaining to their impact on EE (35). However, a number of unexpected findings were unearthed which should be considered in future studies: the influence of expulsion time on the duration of measurement and rapid temperature oscillations, particularly among obese subjects, which require appropriate data treatment to avoid misinterpretation of temperature changes. In addition, the validation study revealed temperature drift, which was not constant and lasted up to 17 h for some capsules. Based on this finding, initial calibration to correct raw data may not negate this issue, as the drift was not always apparent initially or occurred some hours after measurement initiation. Innovation and technical improvement of the capsule will undoubtedly eliminate the drift observed in some of the capsules validated here. Given the renewed interest in Tc as a measure of thrifty metabolism, future studies are warranted to explore the accuracy of Tc measurement *in vivo* using ingestible capsules accounting for the issues highlighted herein.

## Ethics Statement

The study was conducted according to the guidelines laid down in the Declaration of Helsinki and all procedures involving human subjects were approved by the Commission Cantonale D’éthique de la Recherche sur L’être Humain (CER-VD), the ethics committee for human research in the Swiss cantons of Vaud, Fribourg, Neuchâtel and Valais. Written informed consent was obtained from all subjects prior to participation in the study.

## Author Contributions

Conception and design of the work: AD, YS, DD, CM, and JM-C. Data acquisition: CM, JM-C, E-JF, JC, YS, and DD. Data analysis: CM, E-JF, and JC. Interpretation of the data: CM, AD, YS, E-JF, JC, JM-C, J-PM, and DD. Drafted the manuscript: CM and AD. Revised the manuscript: E-JF, JC, JM-C, J-PM, DD, and YS. All the authors approved the final version.

## Conflict of Interest Statement

The authors declare that the research was conducted in the absence of any commercial or financial relationships that could be construed as a potential conflict of interest.
